# Overcoming the Barrier of Low Efficiency during Genetic Transformation of *Streptococcus mitis*

**DOI:** 10.3389/fmicb.2016.01009

**Published:** 2016-07-05

**Authors:** Gabriela Salvadori, Roger Junges, Donald A. Morrison, Fernanda C. Petersen

**Affiliations:** ^1^Department of Oral Biology, Faculty of Dentistry, University of OsloOslo, Norway; ^2^Department of Biological Sciences, College of Liberal Arts and Sciences, University of Illinois at ChicagoChicago, IL, USA

**Keywords:** pheromone, competence, natural transformation, mutagenesis, oral cavity, CSP, streptococcus

## Abstract

**Objective:** *Streptococcus mitis* is a predominant oral colonizer, but difficulties in genetic manipulation of this species have hampered our understanding of the mechanisms it uses for colonization of oral surfaces. The aim of this study was to reveal optimal conditions for natural genetic transformation in *S. mitis* and illustrate its application in direct genome editing.

**Methods:** Luciferase reporter assays were used to assess gene expression of the alternative sigma factor (σ^X^) in combination with natural transformation experiments to evaluate the efficiency by which *S. mitis* activates the competence system and incorporates exogenous DNA. Optimal amounts and sources of donor DNA (chromosomal, amplicon, or replicative plasmid), concentrations of synthetic competence-stimulating peptide, and transformation media were assessed.

**Results:** A semi-defined medium showed much improved results for response to the competence stimulating peptide when compared to rich media. The use of a donor amplicon with large homology flanking regions also provided higher transformation rates. Overall, an increase of transformation efficiencies from 0.001% or less to over 30% was achieved with the developed protocol. We further describe the construction of a markerless mutant based on this high efficiency strategy.

**Conclusion:** We optimized competence development in *S. mitis*, by use of semi-defined medium and appropriate concentrations of synthetic competence factor. Combined with the use of a large amplicon of donor DNA, this method allowed easy and direct editing of the *S. mitis* genome, broadening the spectrum of possible downstream applications of natural transformation in this species.

## Introduction

**FIGURE 1:**
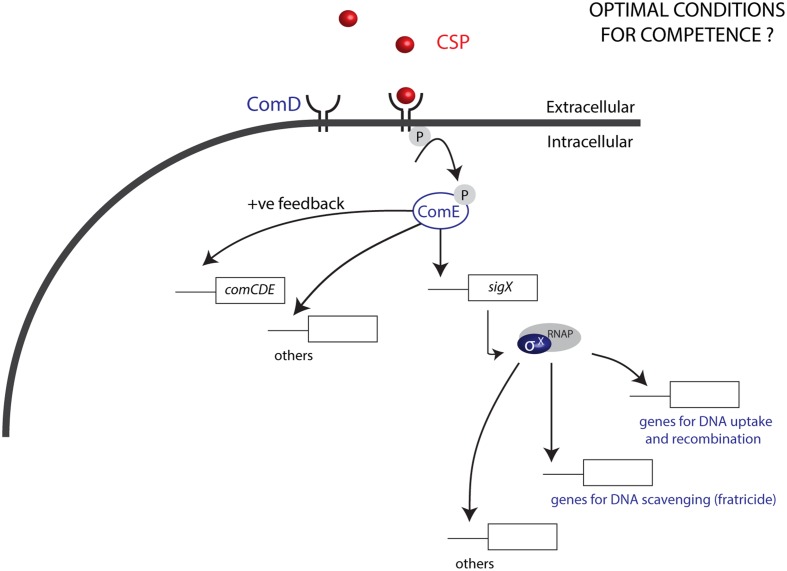
**Competence in the Mitis group of streptococci**. The CSP pheromone produced by streptococci, upon reaching a threshold concentration binds to the histidine kinase ComD, leading to phosphorylation of the response regulator ComE. ComE-P activates a positive feedback loop by inducing the expression of *comCDE*, and also induces the expression of *sigX*, which encodes the alternative sigma factor σ^X^. σ^X^ binds to the RNA polymerase (RNAP), to activate the expression of 27 to 30 panstreptococcal competence genes responsible among other functions for DNA uptake and recombination, as well as DNA scavenging via fratricide. Optimization of conditions for transformation of the Mitis group has been mostly studied in *S. pneumoniae*. For other species in this group, conditions for optimal competence are generally unknown.

Natural transformation, a dominant force in the evolution of bacteria, is responsible for gene exchange and the acquisition of new DNA sequences, and is an important tool in genetic engineering. Most members of the Mitis group of streptococci are naturally competent for genetic transformation and produce competence pheromones, also known as competence stimulating peptides (CSPs), recognized by cognate surface receptors (Håvarstein et al., 1996). Competence development is most studied in *S. pneumoniae*, also a member of the Mitis group. In this model organism, competence is characterized by two main processes: (1) quorum-sensing (QS) and (2) expression of effectors that allow DNA binding, uptake, and integration into the bacterial genome (Claverys and Håvarstein, 2002; Luo and Morrison, 2003). The QS pathway launches the competence development cascade: first, *comC* is activated, and its activation leads to the transcription and translation of the gene into a pro-peptide before it is cleaved and exported as the CSP by an ABC transporter, ComAB (Håvarstein et al., 1996). Once CSP reaches a threshold concentration in the extracellular environment, it binds to its membrane receptor histidine kinase ComD, resulting in the phosphorylation of the response regulator ComE. ComE phosphorylation activates expression of the alternative sigma factor σ^X^ (Lee and Morrison, 1999; Claverys and Håvarstein, 2002) in addition to ∼20 early genes (**Figure 1**). The σ^X^ regulon includes in total 27 to 30 pan-streptococcal core genes (Khan et al., 2016), involved in exogenous DNA uptake, processing, and recombination. Environmental factors are also known to have an important impact on the development of competence and in the magnitude of the response to CSPs. However, in contrast to the molecular mechanisms involved in the CSP response and production that have been described in great detail, such environmental factors are still poorly defined (Johnsborg and Håvarstein, 2009). Notably, while conditions defined for some closely related species can be used as a start point for transformation, optimal levels of competence often require a series of optimization strategies, with an outcome that is not always predictable. In *Streptococcus pyogenes*, for instance, despite a series of optimization approaches, competence has only been demonstrated in the presence of epithelial cells and at levels that do not surpass 0.0001% (Marks et al., 2014).

The human oral commensal *Streptococcus mitis*, a member of the Mitis group of streptococci, colonizes all surfaces of the oral cavity including teeth, tongue and mucosal surfaces, in addition to the tonsils and nasopharynx (Pearce et al., 1995). *S. mitis* and *S. pneumoniae* – one of the most important human pathogens –, share >80% of their genes. Being genetically very similar, their differences in pathogenic potential are striking (Kilian et al., 2014). In comparison to *S. pneumoniae* and their common ancestor, evolutionary analyses suggest that loss of virulence determinants in *S. mitis* may be the reason for the decrease in its pathogenicity (Kilian et al., 2008). In contrast with *S. pneumoniae*, in which most of the strains use one of two pheromones, in *S. mitis* almost each strain has its own CSP (Kilian et al., 2008). Despite the high interest in understanding *S. mitis* behavior, low transformation yields and challenges in genetic manipulation of this species, reflected in the literature for three decades, have probably hampered advances in the field (Gaustad, 1985; Potgieter and Chalkley, 1991; Bensing et al., 2001; Mitchell, 2011; Duran-Pinedo et al., 2014; Rukke et al., 2014; Xie et al., 2015). Prior to the introduction of synthetic CSPs for transformation, Gaustad (Gaustad, 1985) first reported *S. mitis* transformation efficiencies of around 0.0001%. This level was further shown to increase to 0.001% with the use of synthetic CSP by Bensing et al. (2001). Difficulties in transforming *S. mitis* were later also reported for B6, the first genome sequenced strain (Denapaite et al., 2010). For the type strain, Duran-Pinedo et al. (2014) reported failures in obtaining any transformants at all. We have been able to transform the type strain previously (Rukke et al., 2014), but only at low efficiency. Considering the multiple fields in which *S. mitis* has been studied, the development of a highly efficient method for *S. mitis* genetic manipulation through natural transformation is of great interest and value.

We recently described a markerless method for genome editing without introduction of selective markers that is applicable for streptococci with high transformation efficiencies (Morrison et al., 2015). Overcoming the challenges of low transformation efficiencies in *S. mitis* would thus not only facilitate the genetic manipulation of this organism using marker selection methods, but it would also allow the use of direct markerless genome editing approaches in *S. mitis* functional investigations. The aim of this study was to identify optimal conditions for natural genetic transformation in *Streptococcus mitis*, and to illustrate its application as a genome editing resource. We now report the development of a method for high efficiency transformation of the type strain, which is also applicable for strain SK321. Further, we demonstrate its use in direct genome editing.

## Materials and Methods

### Bacterial Strains and Media

All strains of *S. mitis* used are listed in **Table 1**. Streptococci were stored at -80°C in Todd Hewitt Broth (THB, Becton Dickinson and Company, Le Pont de Claix, France) or Tryptic Soy Broth (TSB, Soybean-Casein Digest medium, BactoTM) supplemented with 30% glycerol. Pre-cultures used in the experiments described below were made from fresh liquid cultures incubated at 37°C in a 5% CO_2_-supplemented atmosphere and grown until the cultures reached an absorbance at 600 nm (optical density at 600 nm [OD_600_]; Biophotometer; Eppendorf) of 0.5, before storage at -80°C in 15% glycerol. For transformation assays, Tryptic Soy Broth (TSB, Soybean-Casein Digest medium, BactoTM), THB supplemented with 5% of horse serum (THB-HS) (Petersen and Scheie, 2010), semi-defined media C+Y_Y B_ (Stevens et al., 2011) or C+Y (Martin et al., 1995) were used. For plating, blood agar base No. 2 (Oxoid, Hampshire, England) supplemented with 5% defibrinated sheep blood (TCS Biosciences Ltd., Buckingham, UK) was used. Final concentrations of selective levels of antibiotics were 500 μg mL^-1^ kanamycin, 10 μg mL^-1^ erythromycin, and 200 μg mL^-1^ spectinomycin.

**Table 1:** Strains, synthetic peptides and primers used in this study.

Strains	Description^a^	Source or characteristics
*Streptococcus mitis* CCUG 31611^T^	*S. mitis* biovar 1 type strain, corresponds to NCTC 12261^T^; encapsulated, transformable strain	CCUG


*S. mitis* NCTC 8033 (SK321)	*S. mitis* strain isolated from the oral cavity	NCTC; (Kilian et al., 2008)


*S. mitis* CCUG 62644 (SK575)	*S. mitis* strain isolated from blood; Erm^R^	CCUG; (Kilian et al., 2008)


*S. mitis* CCUG 62641 (SK579)	*S. mitis* strain isolated from blood	CCUG


*S. mitis* CCUG 62642 (SK616)	*S. mitis* strain isolated from blood	CCUG; (Kilian et al., 2008)


MI009	CCUG 31611, but Δ*srtA*::*erm*; Erm^R^	This study


MI055	CCUG 31611, but p*_sigX_* luc::*spc*; Spc^R^	This study


MI074	CCUG 31611, but Δ*SM12261_0092*::*kan*; Kan^R^	This study


MI091	CCUG 31611, but Δ*shp*	This study


MI092	SK321, but p*_sigX_* luc::*spc*; Spc^R^	This study


**Competence Stimulating Peptide (CSP)**	**Sequence**	


*S. mitis* CCUG 31611	EIRQTHNIFFNFFKRR	GenScript


*S. mitis* SK321	ESRLPKIRFDFIFPRKK	GenScript


*S. mitis* SK575	EMRKMNEKSFNFF	GenScript


*S. mitis* SK579	EIRKSNSALVNFFK	GenScript


*S. mitis* SK616	EMRRIDKIFINFLKRR	GenScript


**Primers**	**Sequence (5′ to 3′)**	


FP001^b^	ggcgcgccGTTTGATTTTTAATG	Kan^R^ cassette


FP015^b^	ggcgcgccCCGGGCCCAAAATTTGTTTGAT	Erm^R^ cassette


FP016^c^	ggccggccAGTCGGCAGCGACTCATAGAAT	Erm^R^ cassette


FP068^c^	aggccggccTAGGTACTAAAACAATTCATCCAGTA	Km^R^ cassette


FP411	AGCCGTTCGTGGTATGAGTC	MI009 mutant construction


FP412^b^	ggcgcgccTTTGATACCTGGCTGACTTGG	


FP413^c^	ggccggccAGATCGCGTTGATGAGATTGA	


FP414	CTCAGCCGTTTTTCTCCAAG	


FP770^d^	aaagctagcAGCTGCTTTAGTCGCTGCTC	pFW5-*luc*


FP771^e^	aaaggatccCAATCCCCTGGACTTCTTCA	pFW5-*luc*


FP829^c^	ggccggccTTTTTGCTGTAAACTAAGGAGTAGAGA	MI074 mutant construction


FP830	CGACTTCGATCTTTTAGTTGATG	


FP831	CCCGTTTTCCCAACAAAAA	


FP832^b^	ggcgcgccTTTTCTGTACACCTCATTTCCTTT	


FP914	TCTCGCGGAAGATAAAGTCG	MI009 mutant amplification


FP915	GGGTTAGCAGCAACAAGGAA	


FP1163	CATCTTGATAGCGTGGCTCA	


FP1164	GAAGTCTAAATATGAAGAAGGTGTACACCTAAGTCT	MI091mutant construction


FP1165	AGACTTAGGTGTACACCTTCTTCATATTTAGACTTC	


FP1166	TTGAATTGAGACGGATTGGA	


FP1167	GCTGAGTTCCTCCAAGCTGT	MI009 mutant amplification


FP1168	ATCCAGAAACGACCGACAAG	


FP1169	TCTTTCATCGTGTCCTCCAA	


FP1170	TGATTCTCCCCGTTCAAATC	


FP1171	AAACATTTTATCCACCGACAA	


FP1172	TTGCACCAAGTTAGCGAATG	


FP1242	CTTGAGCTGGGCTTCGTAGT	


FP1243	ACAGGGGATGTCATGGGTAA	


FP1244	CTGCTACGTTTGCAAGGTCA	


FP1245	ATGAACGTGAACCCTTGGTC	


FP1248	CAGCATGCAGTTGCAAAGTT	


FP1249	CCCGAACGTGTTTCTCAAAT	


FP1250	CTCAGCCGTTTTTCTCCAAG	


FP1251	AGTATGGCCGTATTGCCAAG	


FP1280	GCACCTGTATCACGAAGCAA	


FP1281	TGCTGAAGCACAAGCTGAGT	

*^a^Kan, kanamycin; Erm, erythromycin; Spc, spectinomycin.*^b^*AscI restriction site underlined.*^c^*FseI restriction site underlined.*^d^*BamHI restriction site underlined.*^e^*NheI restriction site underlined*.

### Competence-Stimulating Peptide

Stock solutions of the lyophilized CSPs (**Table 1**) were prepared by re-suspending the material in distilled water to a concentration of 10 mM, and storing it at -20°C. Working solutions of 100 μM were aliquoted and stored at -20°C.

### Luciferase Reporter Assays

Culture stocks of the P*sigX*-luciferase strain MI055 and MI092 at OD_600_ 0.5 were diluted in the presence or absence of CSP, and luminescence was monitored as described previously (Morrison et al., 2015).

### Construction of Mutants with Antibiotic Markers

All primers used for marker constructions are listed in **Table 1**. For construction of the *S. mitis* Δ*srtA* (MI009) and Δ*SM12261_0092* (MI074) mutants, the standard PCR ligation mutagenesis strategy was employed (Lau et al., 2002), with minor modifications (Rukke et al., 2014). The *srtA* flanking regions were amplified using primer pairs FP411–FP412 and FP413–FP414. The Δ*SM12261_0092* flanking regions were amplified with the primer pairs FP829–FP830 and FP831–FP832. The erythromycin and kanamycin resistance cassettes (Erm^R^, Km^R^) (Claverys et al., 1995; Sung et al., 2001) were amplified using the primer pairs FP015–FP016 and FP001–FP068, respectively. Ligation and purification of the PCR products were performed using T4 DNA ligase (Fermentas) and the QIAquick PCR purification kit (Qiagen), respectively. The resultant amplicons were used for transformation of *S. mitis* as described below. Correct insertion of erythromycin or kanamycin markers in the deleted region was verified by PCR amplification of the chromosomal DNA extracted from each mutant, using primer pairs complementary to sequences within the antibiotic cassette sequence in combination with primers in regions up and downstream the insertion of the antibiotic cassette. To construct MI055, the luciferase reporter for *sigX*, the primer pair FP770–FP771 was used to amplify the specific promoter region from the *S. mitis* type strain. Following restriction with *Bam*HI and *Nhe*I, the amplicon was ligated to pFW5-*luc* (Spec^R^) and cloned into *Escherichia coli* as previously described (Khan et al., 2012). A plasmid with the correct insert was further purified and transformed into the *S. mitis* type strain, where it was incorporated in the chromosome by single cross-over recombination.

### Transformation Assays

For construction of marker insertions, the following protocol was used. Pre-cultures of the *S. mitis* type strain at OD_600_ 0.5 were diluted 1:10 in TSB and liquid cultures were grown at 37°C, 5% CO_2_ for 15 min. Then, 250 nM CSP was added, together with the PCR constructs or donor plasmids. Cultures were incubated under aerobic conditions at 37°C for 4 h. Transformants were selected on blood agar plates supplemented with the appropriate antibiotics by 24 h of incubation at 37°C, 5% CO_2_.

For optimization steps, different protocols were used as described in the results section.

### Preparation of Plasmids

The replicative shuttle plasmid pVA838 (Macrina et al., 1982) and the non-replicative plasmid pFW5-*luc* (Podbielski et al., 1996) were isolated from *E. coli* using the QIAprep Spin Miniprep kit (Qiagen GmbH, Hilden, Germany), according to the manufacturer’s recommendations.

### Preparation of Genomic Donor DNA

Genomic donor DNA was extracted from strains according to the CTAB method previously described (Petersen et al., 2006).

### Preparation of Donor DNA Amplicons

Primers used for amplification of donor DNA from existing mutants are listed in **Table 1** and corresponding amplicons are described in **Table 2**. PCR conditions and primers can be found elsewhere (Morrison et al., 2015).

**Table 2:** Donor DNA amplicons.

Amplicon	Primers, amplicon size, marker, and template	Source
aRJ19	FP1167/FP1168 – 5.2 kb – Markerless, from MI091	This study
aRJ20	FP914/FP915 – 6.9 kb – Erm^R^, from MI009	This study
aRJ21	FP1163/FP1166 – 6.9 kb – Kan^R^, from MI074	This study
aRJ22	FP1250/FP1251 – 1.9 kb – Erm^R^, from MI009	This study
aRJ23	FP1248/FP1249 – 3.0 kb – Erm^R^, from MI009	This study
aRJ24	FP1280/FP1281 – 3.9 kb – Erm^R^, from MI009	This study
aRJ25	FP1244/FP1245 – 4.9 kb – Erm^R^, from MI009	This study
aRJ26	FP1242/FP1243 – 5.9 kb – Erm^R^, from MI009	This study

### Markerless Mutant Construction

To construct the markerless mutant MI091, with deletion of a putative pheromone gene (*shp*) located between Δ*SM12261_0093* and Δ*SM12261_0094*, two flanking fragments of 3 kb were amplified from *S. mitis* type strain with the primer pairs FP1163–FP1164 and FP1165–FP1166. Overlapping sequences were used for fusion of the two PCR products, in a ligase-free PCR reaction that used less than 50 ng of each of the two amplified sequences and the nested primers FP1167 and FP1168, to produce an amplicon of 5224 bp. After transformation of competent cells of the *S. mitis* type strain, as described in the optimized protocol, colonies from blood plates were screened by PCR, and re-screened to identify a clone containing the correct deletion.

### Detection of Markerless Mutation through Colony Genotyping Assay

Colonies on non-selective plates were screened by PCR using primers specific, respectively, for the donor and recipient alleles at targeted loci, as previously described (Morrison et al., 2015).

### Statistical Analysis

Two-way ANOVA with multiple measures followed by Tukey’s test was used to analyze differences between groups. Student’s *t*-test was used to compare two independent samples. A *p* < 0.05 was considered statistically significant.

## Results and Discussion

### Semi-Defined Medium Enriched with Bovine Serum Albumin Supports High CSP Signaling in *S. mitis*

**FIGURE 2:**
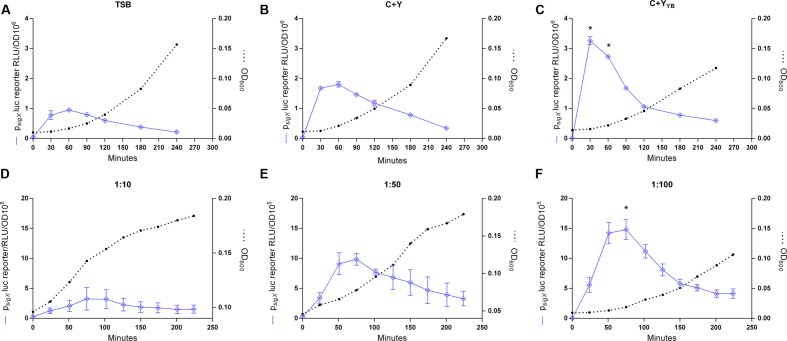
**Effect of growth medium and dilution ratios on CSP-induced *sigX* expression profiles**. **(A–C)** The *sigX* reporter type strain (MI055; p*_sigX_* luc) was grown in 200 μL of TSB, C+Y, or C+Y_Y B_ in a 96-well plate in air at 37°C with 150 nM CSP. ^∗^ Significant statistical difference in C+Y_Y B_ when compared to C+Y and TSB (*p* < 0.05). **(D–F)** Comparison of *sigX* expression profiles and culture growth in pre-culture dilution ratios of 1:10, 1:50, or 1:100. Strain MI055 (*p_sigX_* luc) was grown in 200 μL volumes of C+Y_Y B_ in a 96-well plate in air at 37°C with 150 nM CSP. ^∗^ Significant differences on *sigX* expression comparing 1:10 and 1:50 to 1:100 (*p* < 0.05). For media comparison, pre-cultures were prepared in TSB and on the day of experiment they were centrifuged and re-suspended 1:100 in fresh medium – TSB, C+Y or C+Y_Y B_. For all figures, *sigX* expression, presented as relative luminescence units (RLU), and culture optical density (OD) were measured periodically. The results are averages from 3 replicates and representative of three independent experiments. Error bars represent standard error of the mean. Differences in *sigX* expression among media and dilutions rates were analyzed by two-way ANOVA.

Previous protocols for transformation of *S. mitis* have suggested the use of a rich medium (Todd Hewitt Broth or Tryptic Soya Broth) for culture growth and transformation (Gaustad and Morrison, 1998; Petersen and Scheie, 2010). However, for some species of streptococci, such as *Streptococcus thermophilus*, transformation is mostly restricted to growth in defined media with restricted peptide contents (Gardan et al., 2013). Thus, when looking for alternatives to increase *S. mitis* low transformation efficiency, ranging from 0.001 to 0.5%, we thought to compare competence in TSB with two semi-defined media: C+Y, a medium often used for transformation of *S. pneumoniae*, and C+Y_Y B_, a medium derived from C+Y, with an increased concentration of yeast extract and additional bovine serum albumin, permissive for development of spontaneous competence in *S. pneumoniae* (Stevens et al., 2011). The *sigX* expression was significantly higher in the culture in C+Y_Y B_ medium than in C+Y or TSB, suggesting that this medium might also allow increased rates of transformation in *S. mitis* (**Figures 2A–C**).

### Dilution Rate of Cultures Influences the CSP Response

In *S. pneumoniae*, the basic *in vitro* requirements for high competence to appear include (1) a culture of cells that have not recently been competent since the refractory period, (2) a culture at the early exponential phase of growth, as planktonic cells close to the stationary phase respond poorly to CSP, and (3) exposure to a culture medium conducive to development of competence (Tomasz, 1965; Tomasz and Chambers, 1965; Morrison et al., 1984). The results in **Figure 2** showing background levels of *sigX* expression at the start of the experiments indicate that the cells were not in a competent state, in line with the first requirement for competence in *S. pneumoniae*. As to the second and third requirements, we observed that expression of *sigX* in *S. mitis* is activated early in all three tested media and it is shut off after approximately 30 more minutes (**Figure 2**), and experiments with the addition of CSP to cultures entering stationary phase show a transformation efficiency below 0,001% (data not shown). Our next step was to investigate whether the dilution rate of the pre-cultures into the fresh medium would affect the *S. mitis* CSP response. Although the time to achieve optimal competence levels was not markedly affected, cells from cultures diluted in a 1:100 ratio presented a somewhat (50%) higher *sigX* signaling compared to cells diluted 1:50 or 1:10 (**Figures 2D–F**). Thus, this ratio was used in the further optimization steps described below.

### *S. mitis* Competence for Genetic Transformation is Transient

**FIGURE 3:**
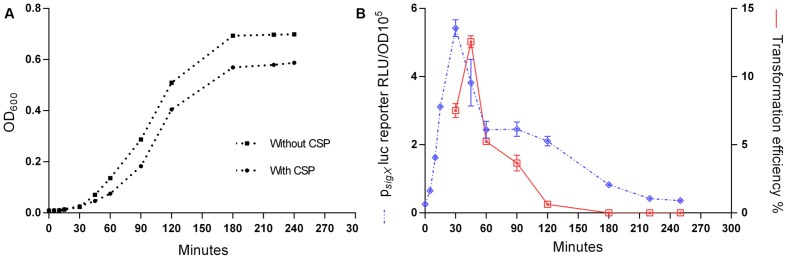
**Responses of the *Streptococcus mitis sigX* reporter in the type strain to CSP**. A pre-culture of MI055 at OD_600_ 0.5 was diluted 1:100 in C+Y_Y B_ and grown until OD_600_ 0.04 at 37°C in 5% CO_2._ At time_0,_ the lag phase cell culture (OD_600_ 0.039) was treated with 300 nM CSP and distributed into 200 μL aliquots that were further exposed to 150 ng mL^-1^ amplicon donor DNA of 7 kb (aRJ20) at different time points. 20 U mL^-1^ DNase I were added after 30 min of exposure to DNA and the culture was incubated in air at 37°C for additional 30 min. The % of CFU containing Erm^R^ transformants was determined by plating on blood plates with or without 20 μg mL^-1^ erythromycin. **(A)** Growth curves with and without CSP, measured as OD_600_. **(B)** Induction of *sigX* and transformation. The results are average from 3 triplicates and representative of two independent experiments. Error bars represent the standard error of the mean.

To explore the kinetics of transformation in *S. mitis*, we performed a time-course study evaluating *sigX* expression employing luciferase reporter assays and monitoring transformation efficiency throughout 240 min of incubation of an inoculum starting with an OD_600_ of 0.04. *sigX* expression increased rapidly after the addition of the synthetic peptide, with its peak occurring at 30 min and decreasing gradually until disappearing at 240 min (**Figure 3**). Following a similar pattern, transformation efficiency reached its highest value (12%) after 45 min of incubation with the competence factor, followed by a gradual decrease. Our results demonstrate that competence for genetic transformation in *S. mitis* type strain is transient, as it is in its close relative *S. pneumoniae* (Håvarstein et al., 1995; Bricker and Camilli, 1999).

### Effect of CSP Concentration on Growth and Transformation

**FIGURE 4:**
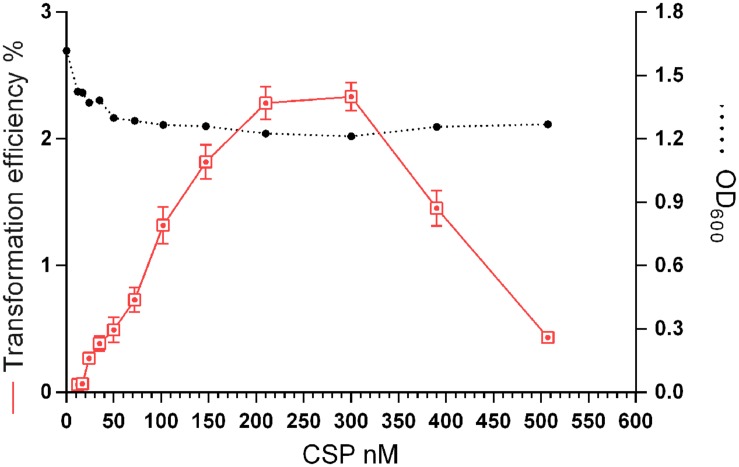
**Effect of CSP concentration on transformation efficiency and culture density**. *S. mitis* type strain pre-culture at OD_600_ 0.5 was diluted 1:100 in C+Y_Y B_ and grown until OD_600_ 0.04 at 37°C in 5% CO_2_. Following, 500 μL aliquots were exposed to 150 ng mL^-1^of the 7 kb amplicon donor (aRJ21) and CSP concentrations varying from 0 to 507 nM. Cultures were further incubated in closed tubes for 4 h at 37°C in air atmosphere. Optical density of the cultures was measured at the time of plating. The % of CFU containing Kan^R^ transformants was determined by plating on blood plates with or without 500 μg mL^-1^ kanamycin. The results are averages from 3 replicates and representative of three independent experiments. Error bars represent standard error of the mean.

In *S. pneumoniae*, cells that are growing rapidly in a competence permissive medium, can be induced to competence by a low (45 nM CSP-1) level of the CSP (Håvarstein et al., 1995). However, a minority of the cells that do not develop competence are lysed by the competent cells, resulting in DNA release (Steinmoen et al., 2002). This fact, along with the previously described CSP-derived stress (Oggioni et al., 2004), may explain the detrimental effect of CSP on *S. pneumoniae* growth rates. Nonetheless, neither the concentration of synthetic CSP for optimal transformation of *S. mitis* nor the effects of the peptide on growth are known. We investigated the transformation efficiency and growth of the *S. mitis* type strain under CSP concentrations ranging from 10 to 510 nM (**Figure 4**). Transformation efficiency was stimulated by a concentration as low as 25 nM, reaching saturation at approximately 210 nM. The final culture density was only slightly affected in the presence of CSP.

### Transformation Efficiency Depends on Amount, Source, and Size of Donor DNA

**FIGURE 5:**
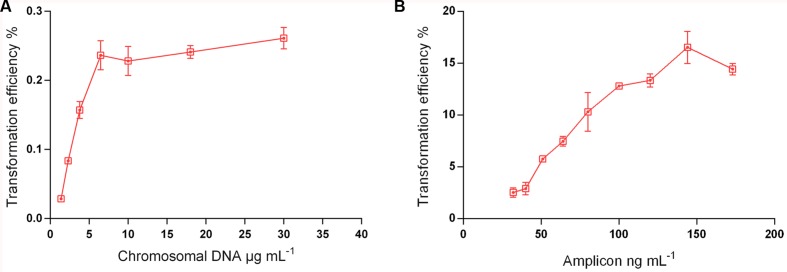
**Transformation as a function of donor DNA type and concentration**. *S. mitis* type strain pre-culture at OD_600_ 0.5 was diluted 1:100 in C+Y_Y B_ and grown until OD_600_ 0.04 at 37°C 5% in CO_2_. **(A)** 200 μL aliquots were exposed to 300 nM of CSP and chromosomal DNA concentrations (from strain MI009) ranging from 1,38 to 30 μg mL^-1^. Cells were further incubated in closed tubes for 3 h at 37°C in air atmosphere. **(B)** 200 μL aliquots were exposed to 300 nM of CSP and 7 kb amplicon (aRJ20) concentrations varying from 32 to 173 ng mL^-1^. Cells were further incubated in closed tubes for 3 h at 37°C in air atmosphere. The % of CFU containing Erm^R^ transformants was determined by plating on blood plates with and without Erm. The results are averages from 3 replicates and representative of three independent experiments. Error bars represent standard error of the mean.

The complexity of the donor DNA is a crucial factor for the yield of recombinants (Morrison et al., 2015). A 7 kb PCR product is, for instance, ∼300-fold less complex than chromosomal DNA from the same genomic template. Thus, chromosomal DNA carries irrelevant genetic material that will compete for incorporation with the fragment of interest. To test this effect in *S. mitis* transformation, we compared chromosomal DNA and a large (7 kb) unique donor DNA amplicon (aRJ20), embodying the same antibiotic resistance insert. **Figure 5** shows that maximum transformation yields were achieved with 6 μg mL^-1^ of chromosomal DNA and 150 ng mL^-1^ amplicon, respectively. These values were close to those previously found in other streptococci, in which saturated concentrations are in the range of 1 to 4 μg mL^-1^ of chromosomal DNA, and 75–100 ng mL^-1^ of amplicon donor DNA (Håvarstein et al., 1995; Bricker and Camilli, 1999; Johnston et al., 2013; Morrison et al., 2015). As expected, the two sources of DNA tested varied in efficiency, with transformation achieving 80-fold higher values with the amplicon than with the chromosomal DNA (**Figure 5**).

**FIGURE 6:**
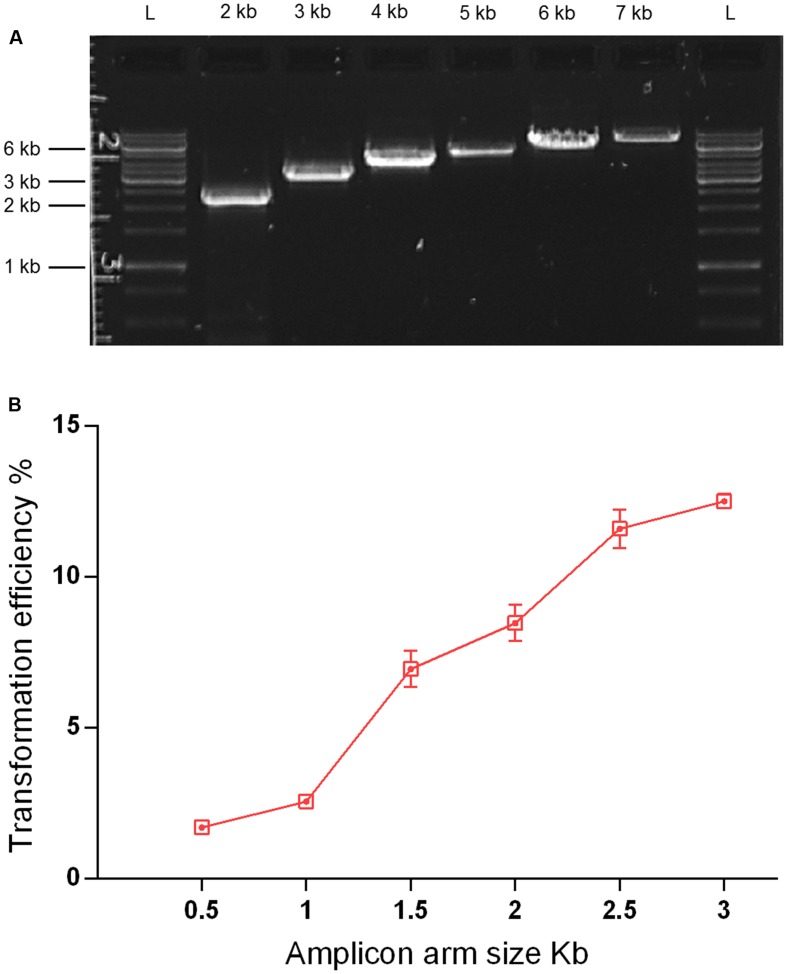
**Effect of amplicon arm size on transformation**. **(A)** Gel analysis of six amplicons of different sizes derived from the same genomic template (MI009). L, DNA ladder Gene Ruler 1kb (GenScript). **(B)** Transformation as a function of amplicon arm sizes. *S. mitis* type strain pre-culture at OD_600_ 0.5 was diluted 1:100 in C+Y_Y B_ and grown until OD_600_ 0.04 at 37°C in 5% CO_2_. Following, 200 μL aliquots were exposed to 300 nM of CSP, 150 ng mL^-1^ donor DNA amplicon (aRJ20, aRJ22, aRJ23, aRJ24, aRJ25, and aRJ26) and further incubated in closed tubes for 4 h at 37°C in air atmosphere. The % of CFU containing Erm^R^ transformants was determined by plating on blood plates with and without Erm. The results are averages from three replicates and representative of three independent experiments. Error bars represent standard error of the mean.

As streptococcal transformation efficiency is strongly dependent on donor DNA fragment size (Cato and Guild, 1968; Morrison et al., 2015), we investigated the effect of amplicon size in the *S. mitis* system. We used unique donor DNA amplicons with a final size from 2 to 7 kb (**Figure 6A**). The amplicons comprised two arms of ∼0.5 to ∼3 kb, flanking a central region with an erythromycin marker. The flanking homologous arms of 1.5 kb increased transformation efficiency by approximately threefold compared to 0.5 kb arms, while further enlargement to 3.5 kb achieved an approximately sixfold increase when compared to 1 kb arms (**Figure 6B**). This is similar to results recently obtained in *S. mutans* (Morrison et al., 2015).

### The Optimized Protocol Results in Transformation Efficiencies Significantly Higher Than the THB-HS Standard Protocol

**FIGURE 7:**
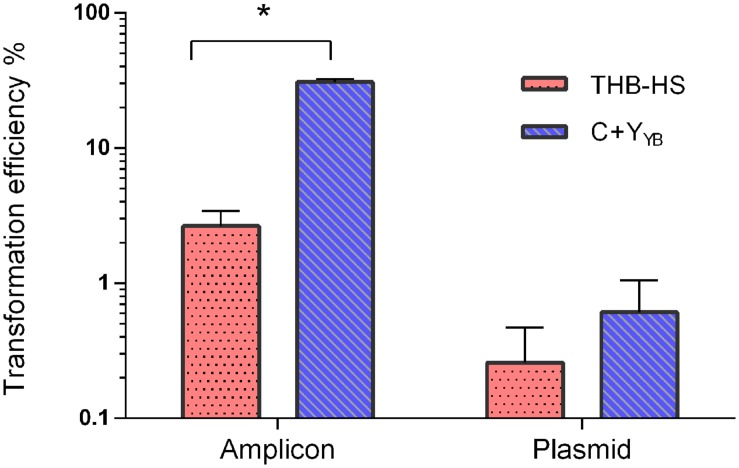
**Transformation as a function of different protocols and donor DNA types**. Following the optimized protocol, *S. mitis* type strain pre-culture at OD_600_ 0.5 was diluted 1:100 in C+Y_Y B_ and grown until OD_600_ 0.04 at 37°C in 5% CO_2_. Then, 200 μL aliquots were exposed to 300 nM of CSP and 150 ng mL^-1^ amplicon (aRJ20) or 1 μg replicative plasmid pVA838. Cells were further incubated in closed tubes for 3 h at 37°C air atmosphere. The % of CFU containing Erm^R^ transformants was determined by plating on blood plates with and without Erm. The results are averages from two independent experiments. Bars represent standard error of the mean. Differences in efficiency between media were analyzed by Student’s *T*-test (^∗^*p* < 0.05).

We compared the optimized protocol in C+Y_Y B_ with previously published protocols in THB-HS (Gaustad and Morrison, 1998; Petersen and Scheie, 2010). The protocol in THB-HS consists of 1:10 dilution of pre-cultures prepared in the same medium, followed by addition of 200 nM CSP and donor DNA, and incubation for 4 h. **Figure 7** shows that the transformation efficiency with pVA838, a second order process, was slightly higher with the new protocol, while transformation with the aRJ20 amplicon as donor DNA resulted in 20-fold higher values, thus confirming the superiority of the optimized protocol in C+Y_Y B_.

### *S. mitis* SK321 Shows Similar Transformation Rates, but Higher *sigX* Expression than the Type Strain

The competence apparatus in *S. mitis* is not always intact, as suggested by a recent study showing that 5 of 15 strains had defects in either *sigX* or other competence effector genes required for transformation (Kilian et al., 2014). This could be one reason why the transformation of *S. mitis* in the laboratory setting has been regarded as challenging. However, with 77% of the strains having apparently intact competence systems, it is expected that a significant range of strains will be amenable to transformation once optimal conditions are defined.

**FIGURE 8:**
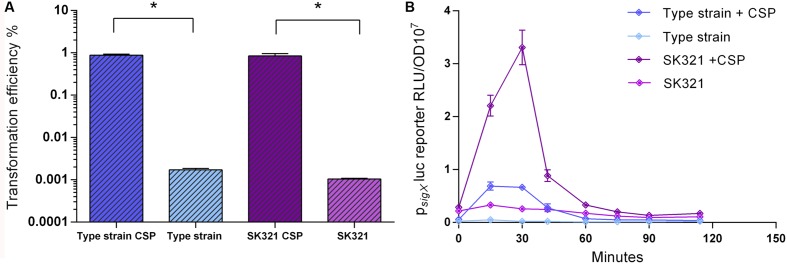
**Comparison of transformation efficiency and *sigX* expression in *S. mitis* type strain and *S. mitis* strain SK321**. **(A)** *S. mitis* type strain and SK321 pre-cultures at OD_600_ 0.5 were diluted 1:100 in C+Y_Y B_ and grown until OD_600_ 0.04 at 37°C in 5% CO_2_. Following, 200 μL aliquots were exposed to 300 nM of their respective strain specific CSP or kept untreated. Cells were transformed with 1 μg mL^-1^ replicative plasmid pVA838 at 37°C in air atmosphere for 3 h. The % of CFU containing Erm^R^ transformants was determined by plating on blood plates with and without Erm. The results are representative of three independent experiments. Bars represent standard error of the mean. Differences in efficiency between cultures with and without CSP were analyzed by Student’s *T*-test (^∗^*p* < 0.05). **(B)** The *sigX* reporter type strain (MI055) and SK321 (MI092) were grown in 200 μL of C+Y_Y B_ in a 96-well plate in air at 37°C with 300 nM of their respective strain specific CSP or kept untreated. Culture optical density (OD_600_) and *sigX* expression (RLU/OD) were monitored periodically. The results are averages from three replicates and representative of three independent experiments. Error bars represent standard error of the mean.

We used the improved transformation protocols for the *S. mitis* type strain presented above to test the transformation of 4 other *S. mitis* strains, using CSPs as deduced from their *comC* sequences. Among these, *S. mitis* SK321 was transformed with pVA838 at efficiencies similar to the type strain (**Figure 8A**), while the remaining strains were not amenable to transformation either with pVA838 or the 7 kb amplicon aRJ21 (percentage similarity of the corresponding region in the reference genomes was approximately 50%). We further constructed a *sigX* reporter in SK321 (MI092) to examine the kinetics of competence development in this strain compared to the type strain. Although *sigX* expression was significantly higher in SK321, the window of competence in the presence of CSP was similar in the two strains, with maximum levels of *sigX* expression between 15 and 30 min (**Figure 8B**).

Analyses of the genomes of these strains available at the Human Oral Microbiome Database^1^ revealed that they possess homologs of all genes known to be involved in development of competence for genetic transformation in *S. pneumoniae*, but with possible truncations or mutations involving the *sigX* gene (**Table 3**). This adds to the body of evidence indicating that some strains of *S. mitis* may not exhibit a fully functional competence apparatus (Kilian et al., 2014). Furthermore, all *S. mitis* strains tested present the constitutively expressed gene *endA*, which encodes for an indispensable endonuclease involved in DNA transport.

**Table 3:** Transformability and putative competence genes in five *S. mitis* strains.

	Genes	*S. mitis* strains^a^				
		CCUG31611^T^	SK321	SK575	SK579	SK616
Transformability		+	+	-	-	-
Early genes	*comAB*	+	+	+	+	+
	*comCDE*	+	+	+	+	+
	*sigX1*	+	+	^∗^	^∗∗^	^∗∗^
	*sigX2*	+	+	-	^∗∗^	-
Core SigX regulon	**Recombination**					
	*radA*	+	+	+	+	+
	*coiA*	+	+	+	+	+
	*dprA*	+	+	+	+	+
	*ssbB*	+	+	+	+	+
	*recA*	+	+	+	+	+
	*comFA-comFC*	+	+	+	+	+
	*cilC*	+	+	+	+	+
	*comEA-comEC*	+	+	+	+	+
	*comGA-comGG*	+	+	+	+	+
	**Fratricide**					
	*cbpD*	+	+	+	+	+
	**Unknown functions**					
	*dut*	+	+	+	+	+
	*rmuC, ccs50*	+	+	+	+	+
	*yhaM, cbf1*	+	+	+	+	+
	*yfiA*	+	+	+	+	+
	*pepF*	+	+	+	+	+
	*pilC*	+	+	+	+	+
	*radC*	+	+	+	+	+
	*cinA*	+	+	+	+	+

^a^*Genome data used for the identification of competence genes: NCTC12261 (CCUG 31611), accession AEDX00000000; SK321, accession AEDT00000000; SK575, accession AICU00000000; SK579, accession AJJL00000000; SK616, accession AICR00000000. ^∗^Substitution of two conserved N-terminal aminoacid residues (E4K; L5M). ^∗∗^Lack of the 5 to 8 C-terminal aminoacid residues possibly essential for proper function of σ^X^. +Present, – Absent*.

### Application for Markerless Transformation of *S. mitis*

**FIGURE 9:**
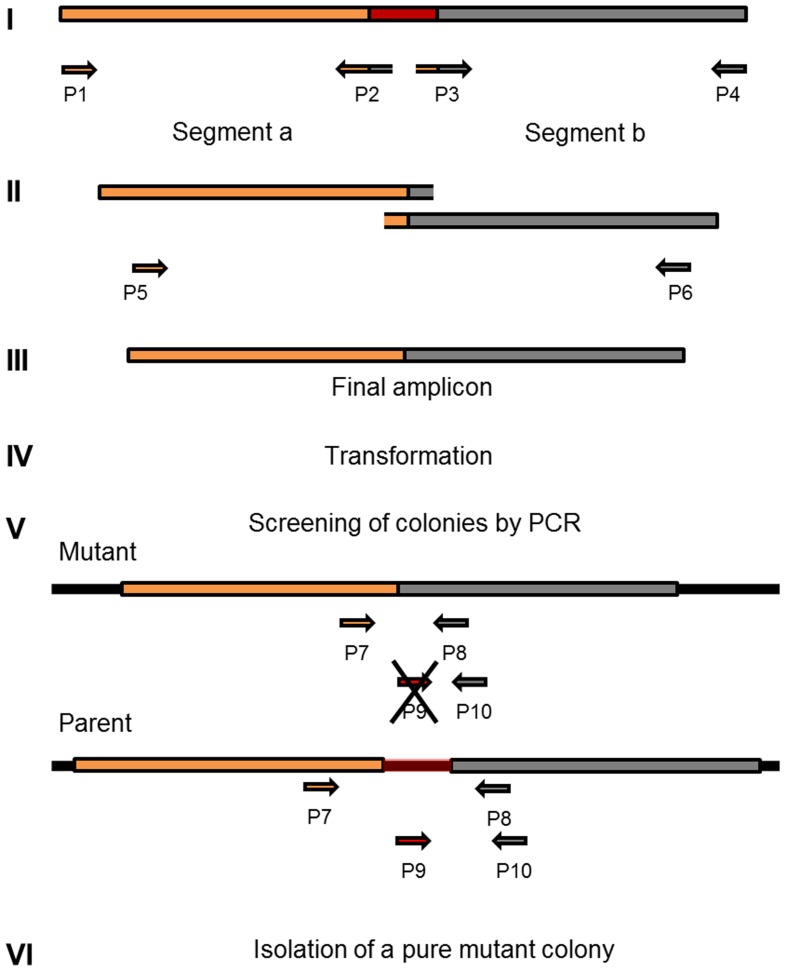
**Workflow for markerless genome editing**. Amplicon design and construction (I to III) are prepared according to the desired mutation, exemplified in this diagram as a gene deletion (marked in red). This step is followed by a highly efficient (>10%) method for natural transformation (IV). Two dozen colonies recovered from the transformation step are screened with specific primers that amplify fragments with different sizes in the mutant when compared to the parent (V). Isolation of colonies with only the new recombined chromosomal DNA (VI).

**FIGURE 10:**
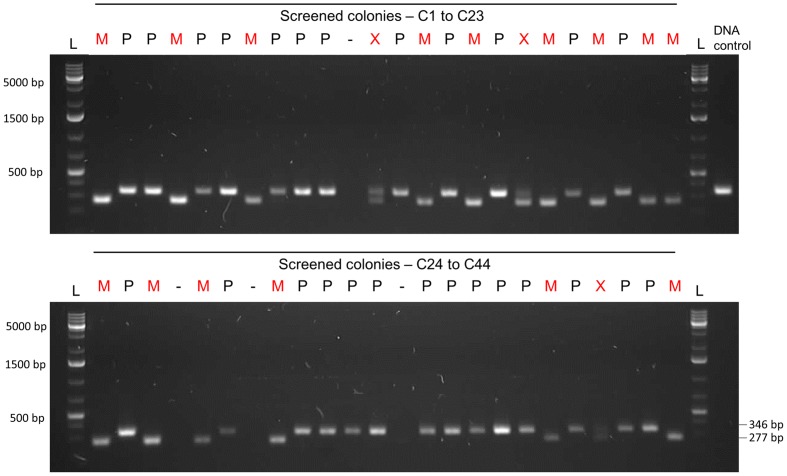
**Gel analysis of the first PCR-screening for the markerless mutation**. Forty-four colonies were screened with pair of primers FP1169/FP1170. Fifteen colonies presented only the mutant allele (M – 277 bp band), while three presented both parent and mutant alleles (X – two bands). Twenty-six colonies presented only the parent allele (P – 346 bp). Transformation efficiency was 41%. L, DNA ladder Gene Ruler 1kb plus (GenScript); DNA control, control with type strain DNA; C, colony followed by the number; M, mutant colony; P, parent strain colony; X. mixed colony presenting both parent and mutant alleles;-, DNA not detected.

**FIGURE 11:**
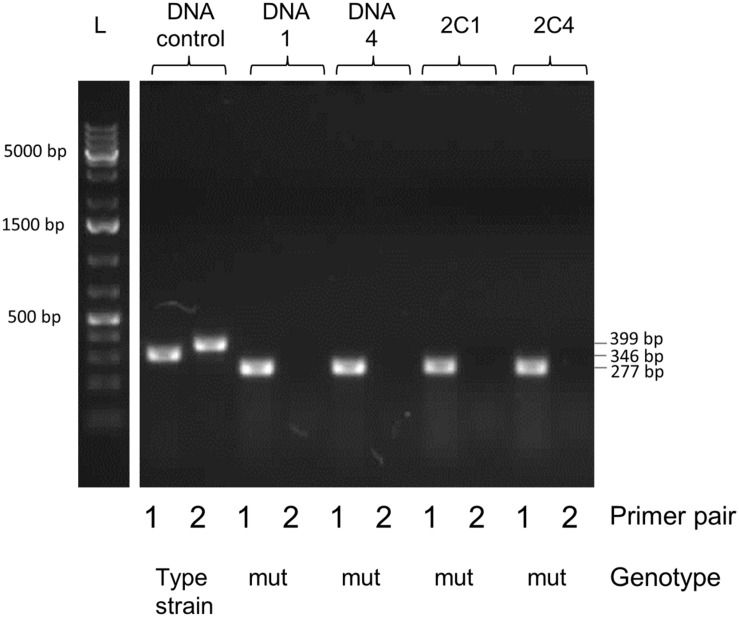
**Gel analysis of segregants from candidate mutant-containing clones**. Primer pair 1 (FP1169/FP1170) amplifies a 346 bp fragment in the parent and a 277 bp fragment in the mutant. Primer pair 2 (FP1171/FP1172) amplifies a 399 bp fragment in the parent but produces no band in the mutant, since primer 1171 is located in the deleted locus. DNA 1 and 4 were extracted from two pure mutant colonies retrieved from the first screening procedure (C1 and C4 in **Figure 10**). Colonies 1 and 4 (C1, C4) were isolated and grown from the first screening procedure for several generations in order to promote segregation of the mutant strain. L, DNA ladder Gene Ruler 1kb plus (GenScript); DNA control, control with type strain DNA; DNA, extracted DNA after the first screening followed by the colony number; 2C, tested colony after growing for several generations to allow segregation, followed by the colony number.

**FIGURE 12:**
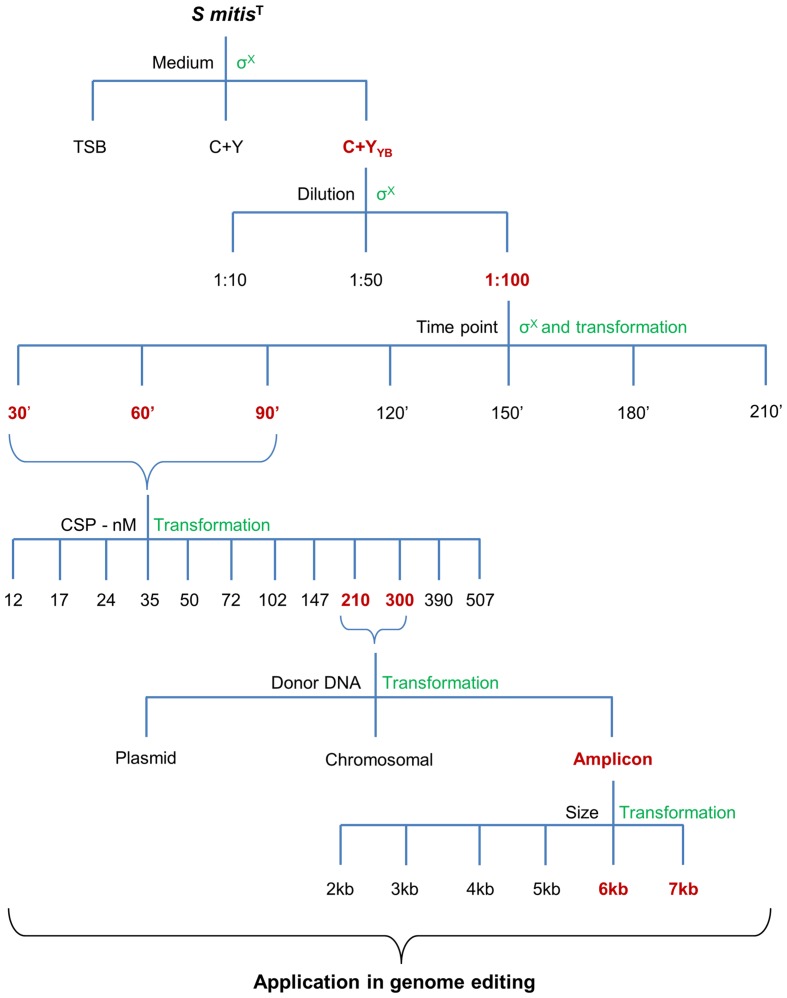
**Summary of all variables tested in the optimization of the protocol**. Different media, culture dilution, time points of transformation, CSP concentration, type of donor DNA, and amplicon sizes were used during the optimization steps. Chosen optimal conditions are in 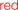, and the methods used to assess competence are in 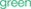.

Selective markers are often used for microbial mutagenesis, especially when transformation efficiencies are low. Despite their value, markers carry unwanted information and might interfere with gene expression or subsequent analysis. The need for multiple mutations may also lead to an accumulation of antibiotic resistance genes. With markerless mutations, it is possible to make as many genome modifications as desired, as long as the mutation is tolerated. In addition, a markerless method based on highly competent cells can be an important tool considering the wide range of possible downstream applications, such as gene knockouts, direct base mutations, promoter editing, and missense mutations. Over the years different markerless mutations strategies have been developed and most of them are based in two-step counter-selection methods (Dailidiene et al., 2006; Xie et al., 2011; Farkas et al., 2012; Thiel et al., 2014). We recently described a one-step method applied in *S. mutans* (Morrison et al., 2015). The high efficiency of our optimized protocol opened the possibility to apply this methodology to create a markerless deletion (69 bp) in an ORF encoding a peptide in the *S. mitis* type strain. Fragments a and b were amplified with primers FP1163/FP1164 and FP1165/FP1166. Both sections were then fused and amplified with nested primers FP1167/FP1168 creating a final donor amplicon of 5.2 kb. This amplicon (aRJ19) carried the 69 bp deletion and ∼2.6 kb flanking regions of homology with the recipient chromosome. The aRJ19 product was used directly to transform *S. mitis* type strain. Forty-four colonies from plates without antibiotics were screened with specific flanking primers for the insertion of the mutation (**Figures 9** and **10**). Forty-one per cent of the colonies contained the mutation allele, among which only 3 colonies (6.8%) presented both the parent and the mutant alleles. Complete segregation was observed using DNA from C1 and C4 (DNA 1 and DNA 4), as well of derived colonies (2C1 and 2C4) that were grown for several generations (**Figure 11**). Thus, we can conclude that segregation took place already within the 3 h of DNA exposure used in the transformation assay. The summary of all variables tested in the optimization of the protocol is presented in **Figure 12**.

## Conclusion

Mechanisms of natural genetic transformation in *S. mitis* have not been thoroughly studied, leaving a gap in the knowledge about this species. As one plausible origin of this lacuna is the challenge in genetically transforming *S. mitis* in the laboratory setting. Thus, the optimized protocol we present here will facilitate future studies of this organism. Based upon the use of a semi-defined medium, specific dilution ratios, increased concentration of CSP, and a large amplicon, we increased *S. mitis* transformation efficiency by more than 3000-fold. This improvement in the protocol allows easy and direct genetic manipulation of the type strain, which also applied to strain SK321, broadening future prospects for the study of genetic transformation in the species as well as of other aspects of *S. miti*s biology.

## Author Contributions

GS, RJ, DM, and FP participated in the conception and design of the work, as well as in the analysis and interpretation of data; GS and RJ conducted the experiments; all authors participated in drafting and revising the work for intellectual content.

## Conflict of Interest Statement

The authors declare that the research was conducted in the absence of any commercial or financial relationships that could be construed as a potential conflict of interest.

## Footnotes

1 www.homd.org
